# NSAID Use Selectively Increases the Risk of Non-Fatal Myocardial Infarction: A Systematic Review of Randomised Trials and Observational Studies

**DOI:** 10.1371/journal.pone.0016780

**Published:** 2011-02-08

**Authors:** Luis Alberto García Rodríguez, Antonio González-Pérez, Héctor Bueno, John Hwa

**Affiliations:** 1 Centro Español de Investigación Farmacoepidemiológica (CEIFE), Madrid, Spain; 2 Departamento de Genómica Estructural, Neocodex SL, Sevilla, Spain; 3 Department of Cardiology, Hospital General Universitario Gregorio Marañón, Madrid, Spain; 4 Section of Cardiovascular Medicine, Department of Medicine, Yale University School of Medicine, New Haven, Connecticut, United States of America; Yale University School of Medicine, United States of America

## Abstract

**Background:**

Recent clinical trials and observational studies have reported increased coronary events associated with non steroidal anti-inflammatory drugs (NSAIDs). There appeared to be a disproportionate increase in non-fatal versus fatal events, however, numbers of fatal events in individual studies were too small, and event rates too low, to be meaningful.

**Objectives:**

We undertook a pooled analysis to investigate the effect of NSAIDs on myocardial infarction (MI) risk with the specific aim to differentiate non-fatal from fatal events.

**Methods:**

We searched Pubmed (January, 1990 to March, 2010) for observational studies and randomised controlled trials that assessed the effect of NSAIDs (traditional or selective COX-2 inhibitors [coxibs]) on MI incidence separately for fatal and non-fatal events. Summary estimates of relative risk (RR) for non-fatal and fatal MIs were calculated with a random effects model.

**Results:**

NSAID therapy carried a RR of 1.30 (95% CI, 1.20–1.41) for non-fatal MI with no effect on fatal MI (RR 1.02, 95% CI, 0.89–1.17) in six observational studies. Overall, the risk increase for non-fatal MI was 25% higher (95% CI, 11%–42%) than for fatal MI. The two studies that included only individuals with prior cardiovascular disease presented risk estimates for non-fatal MI on average 58% greater (95% CI, 26%–98%) than those for fatal MI. In nine randomised controlled trials, all investigating coxibs, the pooled RR estimate for non-fatal MI was 1.61 (95% CI, 1.04–2.50) and 0.86 (95% CI 0.51–1.47) for fatal MIs.

**Conclusions:**

NSAID use increases the risk of non-fatal MI with no substantial effect on fatal events. Such differential effects, with potentially distinct underlying pathology may provide insights into NSAID-induced coronary pathology. We studied the association between the use of nonsteroidal anti-inflammatory drugs (NSAIDs) and the risk of myocardial infarction (MI), separating non-fatal from fatal events, summarizing the evidence from both observational studies and randomised controlled trials. An increased risk of non-fatal MI was clearly found in both types of studies while use of NSAID did not confer an increased risk of fatal MI. Our findings provide support for the concept that thrombi generated under NSAID treatment could be different from spontaneous thrombi.

## Introduction

Non-steroidal antiinflammatory drugs (NSAIDs) are cyclooxygenase inhibitors (COX-1 and −2) used commonly for the treatment of acute and chronic pain. With recent randomised studies demonstrating increased cardiovascular adverse events associated with selective COX-2 inhibitors (coxibs)[1]–[4], there is growing concern and evidence that NSAIDs predispose to myocardial infarction (MI), particularly in those patients at highest cardiovascular risk. The first study to show an increased risk of MI with a coxib was the VIGOR trial [1], which was designed to compare the gastrointestinal safety of rofecoxib and naproxen in patients with rheumatoid arthritis. Although rofecoxib demonstrated a lower risk of gastrointestinal events, there was a four- to five-fold increased risk of MI among users of rofecoxib, but restricted to non fatal events, with apparently no impact on coronary heart disease (CHD) deaths. Due to the low event rate, the clinical significance of this observation was uncertain. With many more studies published since then, there is an opportunity to assess more precisely the relationship between NSAIDs and MI according to its severity. No previous meta-analysis has addressed separately the risk of non-fatal and fatal MI risk associated with NSAID using both RCTs and observational studies.

We performed an analysis of all observational studies and randomized controlled trials published between January 1990 and March 2010 to summarize the impact of NSAIDs on fatal and non-fatal MI in different population types.

## Methods

**Figure 1:**
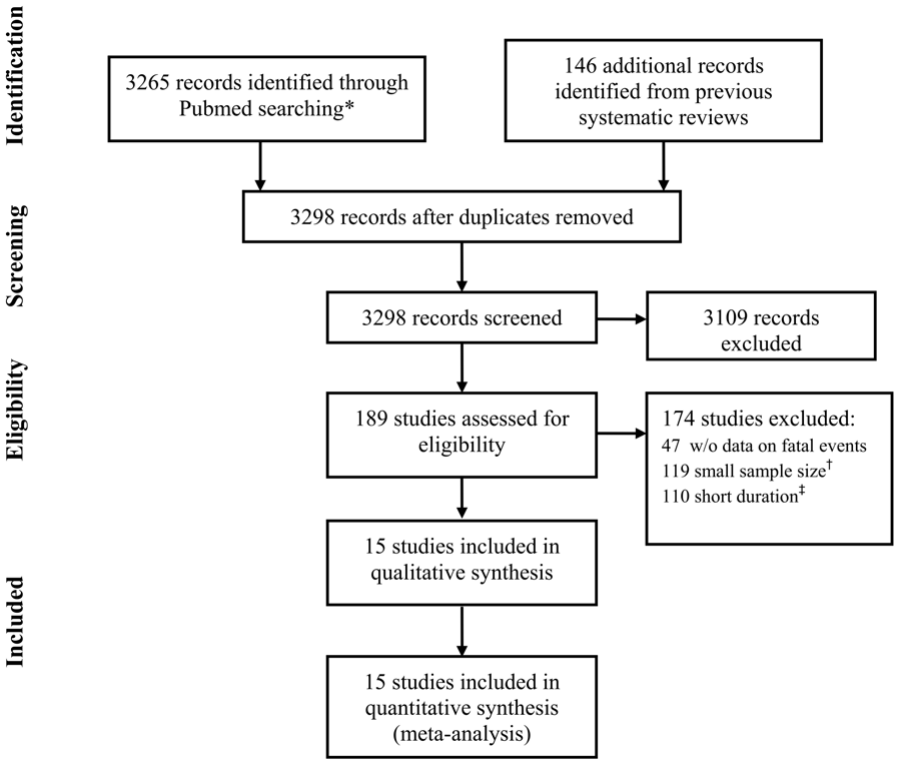
Study selection process.

We identified all observational studies and randomized controlled trials exploring the association between NSAID use and the occurrence of non-fatal MI and fatal-MI (including CHD death), indexed in Pubmed between January 1990 and March 2010 without language restrictions. Additionally we also considered those studies included in previously published systematic reviews. This was particularly useful in capturing data from unpublished RCTs. Studies evaluating traditional NSAIDs (tNSAIDs) and coxibs were considered when they provided either separate estimates of risk for non-fatal MI and fatal-MI according to NSAID use, or sufficient data to compute crude estimates. Because the power of randomised controlled trials to study these rare events is quite limited, we selected only those RCTs with a total sample size greater than 1500, and an average accrued follow-up longer than 6 months. Thus, even though individual studies meeting these sample size and follow-up criteria would still be largely underpowered, assuming an incidence rate of fatal cases around 1.5 per 1000 person-years it would be expected that they found at least one or more of these fatal cases. Additionally, these inclusion criteria would improve homogeneity between studies because only long-term follow-up studies were considered. Data was extracted in duplicate and any discrepancies were resolved through consensus. Extracted data included: type of design, study period, sample size, mean follow-up (in prospective studies), exposure definition, prior history of coronary heart disease (observational studies), use of aspirin (randomized controlled trials), fatal events, non fatal events, and specific estimates for the risk of fatal and non-fatal events. When endpoint specific adjusted estimates were not available and the number of fatal and non-fatal events, as well as the number of exposed and unexposed person-time, were available we estimated the crude incidence rate and relative risk (RR). The detailed results of the study selection process are shown in Figure 1.

In order to obtain weighted summary estimates we used random effects models (Stata 10.0). Among observational studies, we obtained separate pooled estimates according to whether prior history of cardiovascular disease was an eligibility criterion to enter the study. We also obtained separate pooled estimates for randomised controlled trials according to the nature of the comparison group (placebo/NSAID). Finally we explored heterogeneity of effects between non-fatal and fatal endpoints and formally tested this hypothesis by fitting a meta-regression model (stata metareg command). This model provides summary estimates of the relative increase in non-fatal estimates compared to fatal estimates in these studies associated with NSAIDs (odds ratio [OR] and 95% confidence interval [CI]) and the corresponding p values.

## Results

**Figure 2:**
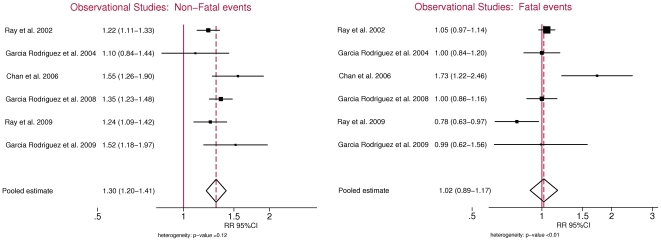
Non steroidal anti-inflammatory drugs and risk for non-fatal and fatal myocardial infarctions in observational studies.

Among observational studies we were able to obtain separate estimates for fatal and non-fatal MI in six studies (Table 1) [5]–[10]. Studies not eligible were those that did not include fatal cases, did not provide separate estimates, or considered as fatal cases all deaths or all cardiovascular deaths. A total of 47 studies were excluded for these reasons. Eligible studies include four cohort studies [5], [7], [9], [10] and two nested case-control studies [6], [8]. The total number of events in these studies ranged from 814 to 8852 non-fatal events and from 277 to 3119 fatal events. The summary OR estimate for fatal and non-fatal MI risk among the 6 eligible studies was 1.21 (95%CI, 1.07–1.37). Using a random effects model, the summary OR estimate for the association between current NSAID use and non-fatal MI was 1.30 (95% CI, 1.20–1.41) compared with 1.02 (95% CI, 0.89–1.17) for fatal MI (Figure 2). Therefore, no association was found between use of NSAIDs and fatal MI. The estimates for non-fatal MI were on average 25% larger than those for fatal MI (OR, 1.25; 95% CI, 1.11–1.42).

**Table 1:** Risk of Non-fatal and Fatal cardiovascular events associated to non steroidal anti-inflammatory drugs in observational studies.

STUDY	Source	Age (years)	Study period	Design	Exposure definition*	Prior CHD (%)	Non-Fatal MI	CHD death
							n_exposed_ (n_total_)	n_exposed_ (n_total_)
Ray ^(5)^	Medicaid	50–84	1987–1998	Cohort	tNSAID 0 days	22	NA (4224)	NA (2138)
Garcia Rodriguez ^(6)^	GPRD	50–84	1997–2000	Nested C-C	tNSAIDs 0–30 days	17	366 (2886)	214 (1909)
Chan ^(7)^	Nurses Health Study	44–69†	1990–2002	Cohort	NSAIDs >22days/month	0	71 (814)	32 (277)
Garcia Rodriguez ^(8)^	THIN	50–84	2000–2005	Nested C-C	NSAIDs 0–6 days	17	901 (8852)	252 (3119)
Ray ^(9)^	Medicaid + Saskatchewan + GPRD	40–89	1999–2004	Cohort‡	NSAID 0 days	100	262 (2484)	96 (1116)
Garcia Rodriguez ^(10)^	THIN	50–84	2000–2007	Cohort§	NSAIDs 0-6 days	100	89 (876)	23 (346)

*Number of days since last use, except in the study by Chan et al in which the current exposure was defined as more than 22 days of use in the last month.

†At baseline (Women only).

‡ Followed up after CHD hospitalization.

§Cohort of aspirin users.

C-C: Case-Control; CHD: Coronary Heart Disease; CI: Confidence Interval; GPRD: General Practitioner Research Database; MI:Myocardial Infarction; NA:Not Available; NSAIDs: non steroidal anti-inflammatory drugs; RR: Relative Risk; THIN: The Health Network; tNSAIDs: traditional NSAIDs.

Two observational studies included only individuals with prior CHD history [9] or prior cardiovascular events (defined as ischaemic heart disease or ischaemic cerebrovascular event)[10]. In this particular subgroup of studies, estimates for non-fatal MI were on average 58% greater than those for fatal MI (OR, 1.58; 95% CI, 1.26–1.98). The remaining four studies included either individuals with no prior history of CHD or a percentage of individuals with prior history below 25%. Among these studies the estimates for non-fatal MIs compared to fatal MIs were of smaller magnitude (OR, 1.19; 95% CI 1.08–1.31).

**Figure 3:**
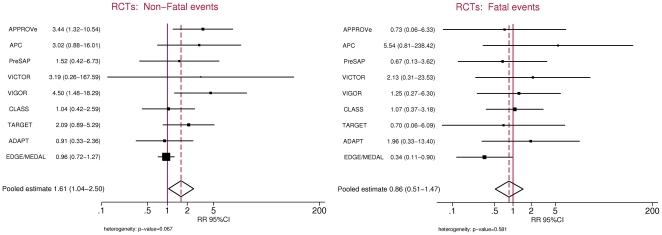
Non steroidal anti-inflammatory drugs and risk for non-fatal and fatal myocardial infarctions in randomised controlled trials.

**Figure 4:**
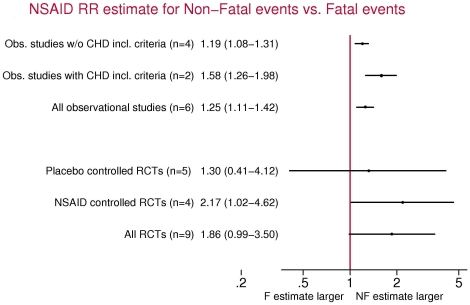
Relative risk estimates for non-fatal compared to fatal myocardial infarction risk associated with use of non steroidal anti-inflammatory drugs by type of study.

Nine randomised controlled trials were eligible for the analysis [1], [2], [11]–[17]. A total of 127 trials were excluded due to small sample size, short follow-up, or both. Sample sizes ranged from 1561 to 34,701 patients whereas average follow-up ranged from 6 months to about 3 years (Table 2). The number of events in individual studies ranged from 4 to 210 for non-fatal MIs and 6 to 23 for fatal events. NSAID treatment arms always included coxibs but the comparator group varied between placebo and tNSAIDs. Overall, the summary estimates for the combined fatal and non-fatal MI risk among the 5 placebo controlled trials and the 4 non placebo-controlled trials were 1.76 (95%CI, 1.09–2.83) and 1.31 (95%CI, 0.78–2.18), respectively. The summary RR estimate for non-fatal MI associated with current coxib use was 1.61 (95%CI, 1.04–2.50) using the random effects model while the corresponding estimate for fatal MI was 0.86 (95%CI, 0.51–1.47) (Figure 3). The estimate for non-fatal MI relative to fatal MI was of borderline statistical significance (OR, 1.86; 95%CI, 0.99–3.50) (Figure 4), although it included both placebo-controlled and tNSAIDs-controlled trials. A secondary analysis restricted to the 5 placebo-controlled trials showed an increased risk for non-fatal MIs (OR, 1.90; 95%CI, 1.05–3.41) but not for fatal events (OR, 1.37; 95%CI, 0.54–3.46). In this subgroup analysis, the OR of non-fatal vs fatal MIs was 1.30 (95% CI, 0.41–4.12). The 4 trials using tNSAIDs as comparators (naproxen, ibuprofen or diclofenac) did not show a significant increased risk of either non-fatal MI (OR, 1.48; 95%CI: 0.80–2.75) or of fatal MI (OR, 0.68; 95%CI, 0.36–1.31) associated with coxib treatment.

**Table 2:** Risk of Non-fatal and Fatal cardiovascular events associated to non steroidal anti-inflammatory drugs in randomised controlled trials.

STUDY	Study Disease	Age (yrs)	Mean Follow-up (yrs)	Exposure	ASA use (%)	Non-Fatal MI		CHD death	
						Tx arm N (%)	Ctrl arm N (%)	Tx arm N (%)	Ctrl arm N (%)
APPROVe ^(2)^	Removed colon adenomas	>40	2.47	rofecoxib 25 mg (n = 1287) vs. placebo (n = 1299)	17–16	19 (1.5%)	6 (0.5%)	2 (0.2%)	3 (0.2%)
APC ^(14)^	Removed colon adenomas	>30	3.01	celecoxib 200 mg bid (n = 685) or celecoxib 400 mg bid (n = 671) vs. placebo (n = 679)	31	18 (1.3%)	3 (0.4%)	11 (0.8%)	1 (0.1%)
PreSAP ^(14)^	Removed colon adenomas	>30	2.5	celecoxib 400 mg (n = 933) vs. placebo (n = 628)	17	9 (1.0%)	4 (0.6%)	4 (0.4%)	4 (0.6%)
VICTOR ^(12)^	Stage II/III colorectal carcinoma	-	0.79	rofecoxib 25 mg (n = 1167) vs. placebo (n = 1160)	8.7–6.9	3 (0.3%)	1 (0.1%)	4 (0.3%)	2 (0.2%)
VIGOR ^(1,11)^	Rheumatoid arthritis	>40	0.57	rofecoxib 50 mg (n = 4047) vs. naproxen 500 mg bid (n = 4029)	4†	18 (0.4%)	4 (0.1%)	5 (0.1%)	4 (0.1%)
CLASS ^(13)^	Rheumatoid arthritis/Osteoarthitis	>18	0.57	celecoxib 400 mg bid (n = 3987) vs. diclofenac 75 mg bid (n = 1996) or ibuprofen 800 mg tid (n = 1985)	22	12 (0.3%)	11 (0.3%)	9 (0.2%)	8 (0.2%)
TARGET ^(16)^	Osteoarthitis	>50	0.74	lumiracoxib 400 mg (n = 9156) vs. naproxen 500 mg bid (n = 4754) or ibuprofen 800 mg tid (n = 4415)	24	18 (0.2%)	9 (0.1%)	2 (0.02%)	3 (0.03%)
ADAPT ^(15)^	Family history of Alzheimer's disease	>70	1.84	celecoxib 200 mg bid (n = 726) vs. placebo (n = 1083)	56	8 (1.1%)	13 (1.2%)	4 (0.6%)	3 (0.3%)
EDGE/MEDAL ^(17)^	Rheumatoid arthritis/Osteoarthitis	>50	1.46	etoricoxib 60–90 mg (n = 17412) vs. diclofenac 150 mg (n = 17289)	34	105 (0.62%)	105 (0.64%)	6 (0.04%)	17 (0.10%)

*Crude Rate Ratios and exact 95% CI are calculated for each study dividing the rate of events in treatment arms over the rate of events in control arms. Crude rates are estimated from the reported number of events and the patient-years of exposure in each arm.

†Prior History of Coronary Heart Disease.

ASA: Acetyl Salicylic Acid (aka aspirin); CHD: Coronary Heart Disease; CI: Confidence Interval; MI:Myocardial Infarction; RR: Relative Risk; Tx:Treatment; Crtl: Control.

Incidence rates of non-fatal MI and CHD death were relatively similar between observational studies and randomised controlled trials. Except one outlier [9], observational studies reported incidence rates between 1 and 8 per 1,000 person-years for non-fatal MI and between 0.3 and 4 for CHD deaths. The corresponding estimates of incidence in randomised controlled trials were between 2 and 5 per 1,000 person-years and 0.4 and 3, respectively.

## Discussion

This pooled analysis reviewing multiple and diverse studies supports the conclusion that NSAID treatment, including both tNSAIDs and coxibs, predisposes to non-fatal myocardial infarction, but only a marginal effect on coronary mortality risk was found. This is important because the elderly population, which has an increased cardiovascular risk, is the major consumer of NSAIDs, so the consistency of our finding of an increased risk of non-fatal MI is of great concern. However, the finding of a lack of coronary-related mortality increase in NSAID users may be considered somewhat reassuring for those who need to be treated with NSAIDs. The hazard of non-fatal cardiovascular events should be taken into consideration when prescribing these drugs in high-risk patients [18].

Based on current knowledge, the underlying pathophysiology of NSAID-associated coronary risk can only be speculated. As patient characteristics are well balanced between patients assigned to the NSAID or control (placebo or active) arms in randomised trials, more advanced coronary disease or other comorbidities (e.g. heart failure, diabetes) are unlikely to be the major explanation for the selective increase in nonfatal cardiovascular events. The differential effects of COX-1 and COX-2 inhibition, regulating thrombus formation [4] is the most plausible mechanism. Selective COX-1 inhibition such as the one produced by low-dose aspirin has been shown to have an antithrombotic effect mediated by a relatively specific, potent, and irreversible inhibition of platelet COX-1, which reduces thromboxane A2 biosynthesis, a powerful trigger of platelet aggregation [19]. Randomised controlled trials with low dose aspirin have consistently shown a clear reduction in risk of non fatal MI and to a lesser extent fatal MI in high risk patients [21]–[25]. In contrast COX-2 inhibition with the resulting suppression of prostacyclin and other hemostatic mediators increase the risk of coronary thrombosis [20]. Thus, an incomplete antithrombotic effect of COX-1 inhibition may counteract the prothrombotic effects of COX-2 inhibition afforded by NSAIDs leading to non-fatal rather than fatal events. A recent study showed that NSAIDs only increase the risk of non-ST-segment elevation acute coronary syndromes but not of ST-segment elevation MI [26]. As the former are related to incomplete occlusion of major coronary arteries or occlusion of smaller coronary arteries [27], it was hypothesized that NSAID-related thrombosis might be less severe than spontaneous coronary thrombosis. Whether coronary thrombi generated under NSAID treatment are different in stability or location from spontaneous thrombi remains to be explored. To directly address the hypothesis that the COX-1 inhibition component of NSAIDs reduces damage caused by MI (shifts the balance towards non fatal MI), the case-fatality effect of NSAIDs individually according to their degree of COX-1 and COX-2 inhibition needs to be assessed. Unfortunately, the current study numbers at the individual level are insufficient to reach any meaningful conclusion.

Only a small number of studies originally reported fatal and non-fatal events separately and met our eligibility criteria. These may not be representative of the majority of published studies in the field. Yet, when we analyzed the combined estimate (fatal and non-fatal MI) in our subset of studies, the results are similar to those reported in previous meta-analyses of observational studies [28], [29] and RCTs [30]. Our review of studies addressing the risk of non-fatal MI and CHD deaths include observational studies, which are susceptible to bias and confounding but based upon the consistency of results across the observational studies and the RCTs, which have well balanced baseline characteristics, it is unlikely that our finding of can be entirely explained by bias. We found some evidence of heterogeneity in our analyses that reached statistical significance for the pooled estimate of fatal events among observational studies (Figure 2). In this context, the use of random effects models is favoured over fixed effects models. Additionally we were able to identify sources of heterogeneity by fitting a metaregression model and exploring the effect of different study characteristics (such as the inclusion criteria in observational studies or the use of placebo in RCTs) on the pooled estimate. Overall, there were insufficient data to assess the risk of non-fatal and fatal MI among individual traditional NSAIDs, or whether there is any difference between traditional NSAIDs and coxibs. This should be the ultimate goal as it has been previously shown that individual NSAIDs at commonly used doses, present variability in their magnitude of risk of ischemic coronary disease [4], [8]. Also, despite our criteria of minimum total sample size and follow-up requirements the power of RCTs to study these rare events is quite limited due to the paucity of events. The inclusion of smaller RCTs with few or no events would provide very little additional information to our risk estimates.

NSAIDs increase the risk of non-fatal MI but do not increase coronary heart disease mortality. Due to the widespread use of NSAIDs in the population, particularly amongst the elderly, further research is urgently needed to unravel the specific mechanisms involved in NSAID-associated coronary thrombosis (including genetic and epigenetic markers) and the identification of patients at highest risk of such complication. Additionally, studies are warranted to reduce the coronary risk associated with NSAID use.

## References

[1] Bombardier C, Laine L, Reicin A, Shapiro D, Burgos-Vargas R (2000). Comparison of upper gastrointestinal toxicity of rofecoxib and naproxen in patients with rheumatoid arthritis. VIGOR Study Group.. N Engl J Med.

[2] Bresalier RS, Sandler RS, Quan H, Bolognese JA, Oxenius B (2005). Cardiovascular events associated with rofecoxib in a colorectal adenoma chemoprevention trial.. N Engl J Med.

[3] Solomon SD, Wittes J, Finn PV, Fowler R, Viner J (2008). Cardiovascular risk of celecoxib in 6 randomized placebo- controlled trials: the cross trial safety analysis.. Circulation.

[4] Grosser T, Fries S, FitzGerald GA (2006). Biological basis for the cardiovascular consequences of COX-2 inhibition: therapeutic challenges and opportunities.. Invest J Clin.

[5] Ray WA, Stein CM, Hall K, Daugherty JR, Griffin MR (2002). Non-steroidal anti-inflammatory drugs and risk of serious coronary heart disease: an observational cohort study.. Lancet.

[6] García Rodríguez LA, Varas-Lorenzo C, Maguire A, González-Pérez A (2004). Nonsteroidal antiinflammatory drugs and the risk of myocardial infarction in the general population.. Circulation.

[7] Chan AT, Manson JE, Albert CM, Chae CU, Rexrode KM (2006). Nonsteroidal antiinflammatory drugs, acetaminophen, and the risk of cardiovascular events.. Circulation.

[8] García Rodríguez LA, Tacconelli S, Patrignani P (2008). Role of dose potency in the prediction of risk of myocardial infarction associated with nonsteroidal anti-inflammatory drugs in the general population.. J Am Coll Cardiol.

[9] Ray WA, Varas-Lorenzo C, Chung CP, Castellsague J, Murray KT (2009). Cardiovascular risks of nonsteroidal antiinflammatory drugs in patients after hospitalization for serious coronary heart disease.. Circ Cardiovasc Qual Outcomes.

[10] García Rodríguez LA, Cea Soriano L, Martín-Merino E, Johansson S (2009). Discontinuation of Low-Dose cetylsalicylic Acid Treatment for Secondary Prevention of Cardiovascular Outcomes is Associated With an Increased Risk of Myocardial Infarction.. Circulation.

[11] Curfman GD, Morrissey S, Drazen JM (2006). Expression of concern reaffirmed.. N Engl J Med.

[12] Kerr DJ, Dunn JA, Langman MJ, Smith JL, Midgley RS (2007). Rofecoxib and cardiovascular adverse events in adjuvant treatment of colorectal cancer.. N Engl J Med.

[13] White WB, Faich G, Whelton A, Maurath C, Ridge NJ (2002). Comparison of thromboembolic events in patients treated with celecoxib, a cyclooxygenase-2 specific inhibitor, versus ibuprofen or diclofenac.. Am J Cardiol.

[14] Solomon SD, Pfeffer MA, McMurray JJ, Fowler R, Finn P (2006). Effect of celecoxib on cardiovascular events and blood pressure in two trials for the prevention of colorectal adenomas.. Circulation.

[15] ADAPT Research Group (2006). Cardiovascular and Cerebrovascular Events in the Randomized, Controlled Alzheimer's Disease Anti-Inflammatory Prevention Trial (ADAPT).. PLOS Clin Trial.

[16] Farkouh ME, Kirshner H, Harrington RA, Ruland S, Verheugt FW (2004). Comparison of lumiracoxib with naproxen and ibuprofen in the Therapeutic Arthritis Research and Gastrointestinal Event Trial (TARGET), cardiovascular outcomes: randomised controlled trial.. Lancet.

[17] Cannon CP, Curtis SP, FitzGerald GA, Krum H, Kaur A (2006). Cardiovascular outcomes with etoricoxib and diclofenac in patients with osteoarthritis and rheumatoid arthritis in the Multinational Etoricoxib and Diclofenac Arthritis Long-term (MEDAL) programme: a randomised comparison.. Lancet.

[18] Bennett JS, Daugherty A, Herrington D, Greenland P, Roberts H (2005). The use of nonsteroidal anti-Inflammatory drugs (NSAIDs): a science advisory from the American Heart Association.. Circulation.

[19] Patrono C, Ciabattoni G, Patrignani P, Pugliese F, Filabozzi P (1985). Clinical pharmacology of platelet cyclooxygenase inhibition.. Circulation.

[20] FitzGerald GA, Patrono C (2001). The coxibs, selective inhibitors of cyclooxygenase-2.. N Engl J Med.

[21] Patrono C, Coller B, FitzGerald GA, Hirsh J, Roth G (2004). Platelet-active drugs: the relationships among dose, effectiveness, and side effects: the Seventh ACCP Conference on Antithrombotic and Thrombolytic Therapy.. Chest.

[22] Antiplatelet Trialists' Collaboration (1994). Collaborative overview of randomised trials of antiplatelet therapy I: prevention of death, myocardial infarction, and stroke by prolonged antiplatelet therapy in various categories of patients.. BMJ.

[23] Antiplatelet Trialists' Collaboration (2002). Prevention of death, myocardial infarction and stroke by antiplatelet therapy in high-risk patients.. BMJ.

[24] Patrono C, García Rodríguez LA, Landolfi R, Baigent C (2005). Low-dose aspirin for the prevention of atherothrombosis.. N Engl J Med.

[25] Baigent C, Blackwell L, Collins R, Emberson J, Antithrombotic Trialists' (ATT) Collaboration (2009). Aspirin in the primary and secondary prevention of vascular disease: collaborative meta-analysis of individual participant data from randomised trials.. Lancet.

[26] Bueno H, Bardají A, Patrignani P, Martín-Merino E, García-Rodríguez LA (2010). Use of non-steroidal antiinflammatory drugs and type-specific risk of acute coronary syndrome.. Am J Cardiol.

[27] Cannon C P, Braunwald E, Libby P, Bonow RO, Mann DL, Zipes DP (2008). – Unstable Angina and Non-ST Elevation Myocardial Infarction.. In Libby: Braunwald's Heart Disease: A textbook of Cardiovascular Medicine, 8^th^ Ed.

[28] Hernández-Díaz S, Varas-Lorenzo C, García Rodríguez LA (2006). Non-steroidal antiinflammatory drugs and the risk of acute myocardial infarction.. Basic Clin Pharmacol Toxicol.

[29] McGettigan P, Henry D (2006). Cardiovascular risk and inhibition of cyclooxygenase: a systematic review of the observational studies of selective and nonselective inhibitors of cyclooxygenase 2.. JAMA.

[30] Kearney PM, Baigent C, Godwin J, Halls H, Emberson JR (2006). Do selective cyclo-oxygenase-2 inhibitors and traditional non-steroidal anti-inflammatory drugs increase the risk of atherothrombosis? Meta-analysis of randomised trials.. BMJ.

